# Steps to Ensure a Successful Implementation of Occupational Health and Safety Interventions at an Organizational Level

**DOI:** 10.3389/fpsyg.2017.02135

**Published:** 2017-12-07

**Authors:** Isabel M. Herrera-Sánchez, José M. León-Pérez, José M. León-Rubio

**Affiliations:** Department of Social Psychology, Universidad de Sevilla, Seville, Spain

**Keywords:** implementation process, intervention evaluation, intervention effectiveness, intervention methodology, occupational health and safety

## Abstract

There is increasing meta-analytic evidence that addresses the positive impact of evidence-based occupational health and safety interventions on employee health and well-being. However, such evidence is less clear when interventions are approached at an organizational level and are aimed at changing organizational policies and processes. Given that occupational health and safety interventions are usually tailored to specific organizational contexts, generalizing and transferring such interventions to other organizations is a complex endeavor. In response, several authors have argued that an evaluation of the implementation process is crucial for assessing the intervention’s effectiveness and for understanding how and why the intervention has been (un)successful. Thus, this paper focuses on the implementation process and attempts to move this field forward by identifying the main factors that contribute toward ensuring a greater success of occupational health and safety interventions conducted at the organizational level. In doing so, we propose some steps that can guide a successful implementation. These implementation steps are illustrated using examples of evidence-based best practices reported in the literature that have described and systematically evaluated the implementation process behind their interventions during the last decade.

## Introduction

An increasing number of evidence-based practices and meta-analysis studies have shown the positive impact of occupational health and safety interventions on employee health and well-being (e.g., van der Klink et al., 2001; Kuoppala et al., 2008; Richardson and Rothstein, 2008; Conn et al., 2009; Martin et al., 2009; Rongen et al., 2013). However, some authors have indicated that the impact of such interventions is limited and inconsistent, particularly when interventions are approached at an organizational level and are aimed at changing organizational policies and processes (e.g., Briner and Reynolds, 1999; Biron et al., 2010; Kahn-Marshall and Gallant, 2012).

Consequently, in recent years several authors have highlighted how failures in the implementation process may explain inconsistent research outcomes when assessing the effectiveness of occupational health and safety interventions (e.g., Nytrø et al., 2000; Saunders et al., 2005; Nielsen et al., 2010a; Nielsen and Randall, 2013), particularly when such interventions are conducted at an organizational level and therefore make it difficult to incorporate experimental designs (Kompier et al., 2000). Intervention implementation in occupational health and safety settings can be defined as the action of transforming and changing the working conditions that affect employee health and well-being in a specific organizational context. Such implementation emerges at the intersection between the design and evaluation of the intervention’s effectiveness. In other words, implementation refers to *what should be done* in order to achieve the desired situation (design) and *what can be done* when taking into account the social reality in which the organization is embedded: its available resources and ongoing results (evaluation). The successful combination of these three elements (design, implementation, evaluation) will most certainly guarantee the effectiveness of the intervention. On the other hand, failures determined by a weak design, inadequate implementation, or poor or non-existent evaluation procedures may explain why a given intervention does not achieve the expected results.

We view implementation as “the dynamic process of adapting the program to the context of action while maintaining the intervention’s core principles” (Herrera-Sánchez et al., 2007, p. 214). Indeed, given that occupational health and safety interventions are usually tailored to specific organizational contexts, generalizing and transferring such interventions to other organizations is a complex endeavor. In response, several authors have argued that implementation evaluation facilitates the early detection of those factors that may moderate or mediate between the intervention’s design and its effectiveness (i.e., results or outcomes), thus allowing researchers to identify those factors that may prove successful under different circumstances when compared to the original context for which the intervention was designed, resulting in major implications for the intervention’s external validity (e.g., Klein and Knight, 2005; Nielsen et al., 2006). In this sense, an evaluation of the implementation process is crucial not only for assessing the intervention’s effectiveness, but also for understanding how and why the intervention has been (un)successful (Nielsen et al., 2010b; Rongen et al., 2013). In other words, implementation evaluation is a prerequisite for being able to explain what actually occurs during the intervention implementation phase and to critically draw conclusions about the effects of the intervention (Dane and Schneider, 1998; Rossi et al., 1999; Nytrø et al., 2000; Nielsen and Randall, 2013).

However, although significant efforts to map the social and cognitive processes influencing intervention implementation in occupational health and safety settings have been made (e.g., Nytrø et al., 2000; Nielsen et al., 2010a; Nielsen and Randall, 2013), some systematic reviews have pointed to a lack of studies examining the relationship between implementation factors and intervention effectiveness (Murta et al., 2007; Egan et al., 2009; Knowlden et al., 2014; Bellicha et al., 2015).

To address this, the current paper focuses on the implementation process and attempts to move this field forward by identifying the main factors that contribute toward ensuring a greater success. In doing so, we propose 10 steps that can lead to successful implementation of interventions at organizational level. These implementation steps are illustrated using examples from previous intervention studies that have described and systematically evaluated the implementation process behind their interventions, which can be considered evidence-based best practices reported in the literature during the last decade.

## Steps To Ensure A Successful Implementation

How to develop and consolidate the effective implementation of an intervention in occupational health and safety settings has been a growing research topic over the last couple of decades. The literature offers general recommendations for implementing interventions (e.g., Fixsen et al., 2005; Meyers et al., 2012); practical guidelines for conducting interventions aimed at improving employee health and well-being at work (e.g., Nytrø et al., 2000; Sheldon, 2007; Weiner et al., 2009; Nielsen et al., 2010a); and even more specific guidelines for implementing certain interventions in organizational contexts, such as those dealing with workplace stress management (Health and Safety Executive, 2007).

Despite these intervention-oriented recommendations, Kompier et al. (2000) pointed out that the success of any intervention depends on the implementation process rather than on the intervention’s own content. Furthermore, they listed the following success factors as relevant to interventions addressing stress prevention at work: a systematic and gradual approach; a proper diagnosis or risk analysis that identifies risk factors and risk groups; a series of theory-driven measures that logically deal with those problems previously identified in the risk analysis; a participatory approach that engages both employees and middle management; and a sustained commitment from senior management. More recently, Nielsen et al. (2010a) revised five standardized occupational health and safety methods for conducting interventions and identified five phases based on the core elements shared by these methods: initiation/preparation, screening, action planning, implementation, and evaluation considering both intervention processes and effects.

Based on these approaches, we turn to previous studies in the field of occupational health and safety (e.g., Biron et al., 2010; Myers et al., 2010; Sørensen and Holman, 2014; Nielsen et al., 2015) to illustrate and describe the key steps that can help guide the implementation and continuous improvement of interventions, as summarized in **Table 1**.

**Table 1:** Main questions an intervention needs to address at each implementation step.

Steps	Questions
(1) Defining the problem	What exactly is the problem?
	Who is exposed to such a problem?
	What are the factors that facilitate (risk) or constrain (protective) the emergence of such a problem?
	What is the perspective of those affected by the problem?
	How does the problem emerge within organizational systems and in their interrelated elements?
(2) Analyzing resources and support	What available resources are needed to deal with the identified problem?
	Where are the resources located and how can they be accessed?
	Which resources have already been mobilized?
(3) Clarifying the goals and objectives	What are the goals and the desired results?
	What are the target groups?
	Is it possible to reach an agreement with stakeholders?
(4) Searching for previous effective interventions	Is it possible to identify other programs that have demonstrated high levels of effectiveness?
	What are the core components of these programs?
	How can these components be adapted to the particular context of intervention?
(5) Clarifying the intervention	What are the core components of the intervention?
	How should these core components fit the needs and problems identified?
	If necessary, what components can be changed to fit the intervention context and which ones cannot?
	How can these components be integrated into the organization?
(6) Promoting team building and empowerment	Which groups may be interested in collaborating?
	How can stakeholders be involved in the process of identifying needs and in selecting and monitoring intervention strategies?
	Is the organization prepared to change its organizational practices?
	Who could be part of the steering committee responsible for the intervention?
(7) Establishing an organizational infrastructure	What are the organization’s underlying values and philosophies?
	Are the intervention objectives and key activities consistent with the organization’s core values?
	What roles and functions are necessary to achieve strong leadership and commitment to change?
	What resources does the organization have to support the intervention?
	Is it possible to consider other organizations in order to achieve the established goals?
(8) Undertaking initial implementation and further development	Does the pilot study confirm the core components identified in the action plan?
	What adaptations are needed following the results of the pilot study?
	How should the intervention elements be sequenced considering the different levels within the organization?
(9) Promoting innovation	How and under what conditions is the program being developed?
	What are its strengths?
	What are its weaknesses?
	What are the results of the program in accordance with the established objectives?
	Which methods and techniques best fit the evaluation questions posed?
(10) Achieving sustainability and integration in standard procedures	How is the intervention more sustainable?
	How can intervention activities be integrated into the organization’s daily routines?
	How can intervention activities be promoted and disseminated?

### Defining the Problem

The first step is to describe and analyze how the problem manifests itself within the organization carrying out the intervention. Thus, the design stage begins by detecting and investigating the problem, taking on board the existing resources to solve it (needs analysis or diagnosis: Herrera-Sánchez et al., 2006). This implies giving a working definition of the problem and its evaluation in the context in which the problem occurs.

According to Kelly et al. (2000), intervening from an ecological perspective requires a vision of how people and their social systems affect each other, and not just an examination of their independent qualities. This is the case of the ‘Stand Up Australia’ intervention (Neuhaus et al., 2014), which describes best practices to promote occupational health and safety from a multi-component approach that addresses the multiple intertwined influences of the political, physical and psychosocial environment on individuals’ behaviors. Thus, the organizations’ characteristics together with how their corresponding systems interrelate may determine the feasibility and appropriateness of adopting certain strategies. In this sense, Biron et al. (2010) showed how, even when an organization decides to adopt stress prevention initiatives in line with mandatory legislation, certain context-specific circumstances may lead to poor implementation, which, in turn, may negatively affect its results. For example, the decision to adopt participatory strategies using a workshop format, which provides a space for constructive dialog, may work in contexts where there is a low power distance between workers and managers; however, this type of strategy may not prove successful in more authoritarian contexts, or where conflicts arise between employees and management (Nielsen et al., 2015).

Thus, the tasks to be performed at this initial stage include: (a) identifying the main problem and the most vulnerable groups; (b) analyzing both the risk and protective factors associated with the problem; (c) examining the ecological environment and its interdependent systems to determine how they affect the problem; (d) examining the different levels of intervention that fit with the organization (Nielsen et al., 2010b); and (e) addressing how the organizational culture and values condition employee perceptions and health behaviors. In addition, it is important to tackle the problem by taking the points of views of those involved or aware of the situation into account.

Different sources of information should be collated at this stage, ranging from literature reviews and conceptual analyses of the problem to consultations with key groups to gain access to records that contain statistical data on the problem. The more varied the information collected, the better placed one is to set realistic goals suited to the identified needs, establishing criteria for deciding which groups the intervention should focus on, and gathering data to log the changes introduced by the intervention. For example, Sørensen and Holman (2014) opted for a workshop-based, participative intervention aimed at diagnosing problems and developing change initiatives, focusing on organizational change and job redesign. This type of realistic, context-specific information helps those groups with a potential role in developing the program become aware of the need to intervene, meaning that the intervention receives the support it needs to be a success.

### Analyzing Resources and Support

Along with defining the problem, it is important to examine the available resources and support that could help mitigate the risk conditions or enhance protective factors. This is particularly relevant for small organizations where resources are more limited. Identifying such resources provides insight into issues that are already being addressed as opposed to those not yet on the agenda, thus avoiding duplication of effort. In addition to established programs and services, it is useful to identify those services that can lend intervention support through the provision of funds, spaces and other resources.

In this step, intervention designers gather information on the location, accessibility and amount of available resources (Chinman et al., 2004) by holding meetings with all social agents (managers, middle management, representatives) to ensure that the intervention fits with the organizational context and that the existing resources cover all phases of the intervention. For example, Biron et al. (2010) described a case in which the greatest efforts in terms of resources and time were directed toward the design of sophisticated tools for risk assessment. However, these tools ended up not being used during the implementation phase.

### Clarifying the Goals and Objectives

Having identified the need for intervention and available resources, the next key step is to specify the goals of the program, the target population, and the desired outcomes. On the premise of cooperation, all groups involved need to reach a consensus concerning the project objectives. The same groups that had previously discussed the problem can also set the intervention objectives. Thus, in this stage, goals should be articulated and described in a clear and direct manner. Once participants agree on the objectives, they can turn to the decision-making process. Here they select the theory-based model that will guide the intervention; outline the implementation details; and discuss how to mobilize resources, measure the effects of the program, and respond to criticism and manage resistance to change. For example, Nielsen et al. (2015) centered their intervention on workshop sessions involving all health and safety members within the organization (health and safety representatives, supervisors, managers, senior management), establishing specific intervention areas that emphasize workplace safety. These workshops yielded detailed agreements on specific activities to be carried out (e.g., developing and implementing new safety procedures).

### Searching for Previous Effective Interventions

It is important to look for evidence-based interventions that respond more adequately to the goals and objectives identified by and negotiated with the groups involved in our own intervention. Such evidence-based interventions are often categorized as *best practices*, that is, interventions that have consistently shown positive outcomes through a rigorous evaluation of their processes and results. Adopting these best practices implies determining not only how they fit with the goals and objectives of our intervention, but also how these previous interventions fit with the social ecology underlying our intervention context (Chinman et al., 2004). For example, Nielsen et al. (2015) based their workplace safety management intervention on DeJoy’s (2005) intervention strategy which activates participatory problem-solving processes as well as culture change, and which has shown to be effective. However, its limited duration (under 26 weeks), together with the inherent characteristics of Nielsen and colleagues’ intervention in small enterprises, required an adaptation of outcome measures. Instead of measuring culture change, the authors focused on a more specific operationalization of the “safety levels” construct, which comprised culture-oriented (e.g., leadership, knowledge, involvement); structural (health and safety representatives’ commitment); and behavioral measures (safety behavior).

### Clarifying the Intervention

Here implementation is viewed as the dynamic process of adapting the intervention to the performance context while maintaining its core principles (adaptation vs. fidelity, e.g., Randall et al., 2005; Egan et al., 2009; Augustsson et al., 2015). In this step, the main and difficult tasks are, on the one hand, to identify which intervention components should remain unchanged (i.e., the most essential and indispensable components for maintaining the intervention’s identity and effectiveness) and, on the other hand, to identify which components should be adapted to fit with the social ecology under the new intervention scenario, but without affecting its effectiveness (Dalton et al., 2001).

In doing so, it is necessary to conduct a systematic replication of the intervention; the assumptions and mechanisms that explain how and why the intervention might achieve improved working conditions should be clearly indicated. The intervention’s underlying theory should help to maintain the principles of effectiveness identified in the original intervention (core components) and should therefore increase the likelihood of delivering similar results to those achieved by the original intervention. In short, the task of identifying or developing an intervention theory is crucial during this stage (Bickman, 1987; Chen, 1990; Rossi et al., 1999).

Moreover, intervention adaptation is required here. According to Hunt et al. (2007), the elements at the original intervention’s surface and deep structure levels should be adapted by taking into account the identified needs and problems, the intervention’s social ecology, and the cultural factors/socioeconomic characteristics of the target group(s). For example, in the case of an intervention addressing safety behavior, the core components are clearly identified in the literature based on the principles of behavioral analysis, but require adjustment to a variety of work settings such as hospitals, offices, transportation, mining and factories (Myers et al., 2010).

Finally, it is necessary to introduce the appropriate methods and procedures to determine implementation fidelity (implementation evaluation) and to study the effects of the intervention. As such, an implementation manual would benefit all concerned, including guidelines or instructions on how to implement each activity and the required support materials and resources. These instructions can be perfectly embedded into the intervention theory as illustrated by Biron et al. (2010, see p. 140). These authors provide a figure which outlines the underlying assumptions for the intervention that the managers should be aware of for the program to work. In short, it is crucial that this step defines an action plan that clearly describes the program’s objectives, deadlines and each proposed change initiative and the people responsible for conducting such initiative, as well as its success criteria (Nielsen et al., 2010a).

### Promoting Team Building and Empowerment

Change can be initiated and occurs when an organization and its members demonstrate awareness, commitment, and action capacity. From this perspective, several authors have attributed the success of their interventions in the occupational health and safety context to the participation and involvement of stakeholders, managers, and employees (Nytrø et al., 2000; Hunt et al., 2007; Nielsen and Randall, 2013; Nielsen et al., 2015; León-Pérez et al., 2016). According to Weiner et al. (2009, p. 294), “implementation activities […] must be coordinated and synchronized for employees working in different functional departments, work shifts and work locations.” A comprehensive occupational health and safety intervention must be understood as an innovation within the organization and, as such, requires a “collective behavior” that drives forward change. This would bring about collective benefits for the organization such as improved health, greater productivity, and reduced healthcare costs (see León-Pérez et al., 2016).

In other words, workers and social agents’ involvement is necessary to create favorable and optimal conditions to enable the desired change. For example, not only do they play a key role in guaranteeing that the implementation activities fit with the needs and values of the groups involved in the intervention, but they are also well positioned to anticipate and address any potential resistance to change. Moreover, given that organizational interventions usually involve some kind of change within the organization, stakeholder participation is needed to be able to handle these changes and avoid resistance (Mackay et al., 2004; Nielsen et al., 2010a). The greater the groups’ involvement and participation, the greater the likelihood of achieving a sense of ownership which can lead to a lasting and sustained commitment.

These groups should play an active role in recognizing their needs and resources, selecting strategies and services, monitoring and following up on interventions and, finally, supporting intervention sustainability. Different experiences of collaborative intervention in the organizational context have yielded positive results (McVicar et al., 2013; Sørensen and Holman, 2014). In terms of effective participation, certain conditions and activities are required, starting with identifying the groups that may be interested in collaborating. In addition to encouraging work team participation, other influential groups (middle and senior management) can help when it comes to obtaining the necessary support. Moreover, it is important to ascertain the organization’s willingness to adopt an intervention and work toward strengthening their capacity to implement such programs. Organizational disposition and readiness for change refers to the extent to which implementing employees “are psychologically and behaviorally prepared to make the changes in organizational policies and practices that are necessary to put the innovation into practice and to support innovation use” (Weiner et al., 2009, p. 296).

As for coordinating organizational participation, specific steering committees are often set up to solve problems, as well as focus groups whose job entails identifying needs, assessing risks, and voicing suggestions for improvement. Myers et al. (2010) documented how different safety committees were established whose main purpose was to promote communication between the different safety areas. Sørensen and Holman (2014) developed workshops made up of managers and employees to ensure that the intervention fit with the organization and its people. During these workshops, the most salient aspects of work and well-being capable of prioritizing change initiatives were highlighted. Both authors observed how the employees’ main concerns were more about developing initiatives such as leader feedback and knowledge sharing to reduce ambiguity and uncertainty than about other activities that had been identified in the literature as key components for improving employee well-being, such as increasing task control and task variety. Finally, León-Pérez et al. (2016) conducted conflict management training at a healthcare organization in which they “also trained the department’s line managers to gain their support and ensure their involvement with the intervention.” (p. 4).

### Establishing an Organizational Infrastructure

We can state conclusively that interventions heavily depend upon the degree of responsiveness shown by the organizations that promote them. Therefore, for an intervention to be successful, besides addressing its design aspects (intervention theory), an organizational context must be developed in accordance with the intervention requirements. Mellor et al. (2011) examined 100 public sector organizations that implemented work-related stress prevention and reduction guidelines in line with the “UK Management Standards” that focus on risk assessment and management, covering the 2007–2009 period. They found that continuous processes of change within an organization, a goal-oriented culture, and lack of support from senior management can interfere with an intervention’s progress. As a result, it may be concluded that intervention success is, to a large extent, dependent on how well managerial culture, based on quality, effectiveness and efficiency, is promoted.

In other words, this step is about having the resources and means for implementing and sustaining the intervention at one’s disposal. This requires: (a) commitment from the organization and its members to adopt the intervention; (b) the establishment of clearly defined roles and functions of strong leadership; (c) staff committed to the intervention and, where appropriate, a plan for staff selection and training; (d) the necessary materials and financial resources or a plan on how to obtain them; and (e) establishing connections with other organizations. Ultimately, where possible, interventions must be carried out in organizations that are trained to implement them.

In this regard, and as a key point, it is important to ensure that the program objectives tie in with the aims and goals of the organization hosting the intervention. As Dalton et al. (2001) indicated, it is unlikely that an organization will adopt a prevention or health promotion initiative unless its members are able to establish a clear relationship between the purposes of the initiative and the organization’s mission. Thus, a workplace stress prevention intervention would only work if management were open to structural changes if required. Specifically, Myers et al. (2010) documented an experience in behavioral safety where the alignment of the organization’s values and objectives, together with the identification of relevant practices that support them, constituted the essence of the intervention. The management team had a clear mission to reduce potential harm to employee health and well-being and therefore shifted the culture toward workers’ health and safety values. Olsen et al. (2009) introduced a safety intervention into a process of organizational change in which management involvement and commitment were considered one of its success factors. From this perspective, for an intervention to be accepted and supported, all parties must: (a) examine the organization’s underlying values and philosophies through its plans and action strategies; (b) analyze the intervention’s objectives and key activities to determine to what extent they are consistent with the organization’s core values and, if needed, specify the necessary modifications and adaptations; and (c) strengthen the leadership committed to these values and, if appropriate, redirect them so that they are compatible with the intervention’s goals and objectives.

### Undertaking Initial Implementation and Further Development

Implementation implies somewhat drastic changes for an individual or organization (skills, organizational capacity, political strategy, etc.). However, these changes take time to develop and consolidate; meanwhile, trust and sense of ownership toward the intervention increases. Thus, as reported by Fixsen et al. (2005), these changes do not occur simultaneously within an organization; the various intervention components need to be implemented sequentially. From this perspective, the intervention process can begin with a pilot study aimed at achieving a few goals, but confirming the principles and core components of the intervention which will undergo fidelity replication. The results of this pilot study may facilitate a careful analysis of the effects of the current intervention context, therefore encouraging dialog on the need to modify and adapt said intervention. Hence, potential modifications are made on the basis of a research-action process where implementation is monitored, the results are analyzed, feedback is provided, and intervention adjustments and adaptations are examined and discussed.

To summarize, a pilot study enables social agents leading the intervention to become familiar with the content and materials before the program officially starts, which helps to define intervention adjustments (intervention clarification) as well as to organize the available resources (organizational infrastructure). For example, Neuhaus et al. (2014) introduced two pilot studies to determine the effectiveness (efficacy), viability (feasibility) and acceptability of the intervention, which served to identify its core components. Similarly, Myers et al. (2010) launched a pilot study in an area of a company with a high incident rate before introducing their safety management intervention in a petroleum refinery.

Once adaptations to the current context have been considered, and taking into account that interventions at an organizational level entail multiple actions at various levels (e.g., individual, interpersonal, organizational), the intervention components can be implemented sequentially. Indeed, an intervention is fully implemented when the organization adopts the policies, procedures, and resources required for its implementation; the team of professionals involved acquires the skills and abilities necessary for its implementation; and when the entire organization knows the intervention and adapts itself to it (Fixsen et al., 2005). In a similar Vein, Sørensen and Holman (2014) differentiated between high-, medium- and non-implementing organizations depending on the type, extent and speed of initiative implementation in accordance with the change initiatives planned during the intervention design phase. The conclusion drawn from their process evaluation was that the degree of implementation affected the results of the six examined interventions aimed at improving working conditions and psychological well-being in Denmark. In high-implementation organizations, employees reported greater activity involvement, increased support from their superiors, and more information about the intervention compared to their peers in organizations that fall under medium- and non-implementing groups. Furthermore, employees working for organizations classified as high in terms of intervention implementation reported significant higher improvements in work relationship quality and greater reductions in burnout.

### Promoting Innovation

The value of an intervention lies in how well it is able to adapt existing empirical evidence to the emerging and different circumstances in which the intervention takes place. Thus, in this step we focus on introducing evaluation mechanisms that can be used as a tool for ongoing learning and improving the intervention during the implementation phase (process evaluation), as well as an indicator of intervention results and effects (outcome evaluation).

Process (implementation) evaluation is about monitoring and assessing the different intervention activities in order to identify the strengths and weaknesses that provide useful feedback for improvement. In this sense, process evaluation requires all parties involved in intervention implementation to participate in the decision-making and problem-solving processes. As a result, this evaluation may help explain the results obtained following intervention, or at least how and under what boundary conditions can an intervention succeed or fail. Furthermore, there are interventions with similar objectives that can give rise to different or unexpected effects within a certain context that only process evaluation can help understand (Nielsen et al., 2006). In addition, process evaluation is needed to determine the potential for moving the intervention from its originally intended context, for which it was designed and implemented, to a different one. Along these lines, Nielsen and Randall (2013) proposed a model for evaluating occupational health interventions at an organizational level which they felt encompassed those mechanisms capable of linking intervention processes with their outcomes. They developed this evaluation model based on three key pillars: intervention design and implementation, the intervention context and its ecological validity, and the participants’ mental models of the intervention and its subsequent impact on behavior change. Recently, Augustsson et al. (2015) showed how some of these factors (context, intervention, mental models) seem to explain variations in implementation between units within the same organization. Even in the case of organizational interventions, where effectiveness is difficult to determine, it is reasonable to assume that the main indicator of success is an intervention subject to a continuous improvement process rather than one that obtains isolated positive results.

On the other hand, outcome evaluation focuses on assessing whether or not the program has achieved the proposed and desired goals (e.g., improving employee health and well-being). This evaluation type frequently seeks to obtain information about the intervention’s efficacy, efficiency, and effectiveness that serves decision making and future planning: discarding actions that have proven ineffective and returning to those that have been a success.

In this regard, this type of evaluation requires an objective, independent evaluator in a position to take full responsibility for the evaluation process, as opposed to stakeholders and other social agents who may have interests beyond the intervention goals. As for evaluation methodology, all options are considered valid as long as they meet the needs of the intervention and the evaluator (Herrera-Sánchez et al., 2005). In the early stages, when little is known about the problem and its potential solutions, it is useful to explore and describe the intervention’s unique features (for example, through case studies). When an intervention is sufficiently implemented locally, thus making it possible to evaluate its effectiveness, the evaluation process requires causal or probabilistic explanations (correlational, experimental or quasi-experimental designs). Meanwhile, when similar interventions across different organizational contexts are at play and policy makers wish to gather evidence about which intervention is more generalizable, evaluation should be based on a systematic literature review and synthesis or meta-analysis. Finally, the intervention evaluation process may go beyond evaluating the intervention’s impact by also considering the value of various alternatives for solving social problems, leading to interpretive evaluations using the hermeneutical approach.

Regarding occupational health and safety interventions at an organizational level, mixed-method evaluation designs undoubtedly stand out as the most appropriate methodology when it comes to determining an intervention’s effectiveness, while also providing a broad overview of the whole implementation process (Lipsey and Cordray, 2000; Nielsen et al., 2010a; León-Pérez et al., 2012; Jenny et al., 2015; Abildgaard et al., 2016). For example, Sørensen and Holman (2014) applied a longitudinal design combining qualitative and quantitative methods to examine the processes and results of an intervention to improve working conditions and employee health at six organizations in Denmark. During the 14-month-long intervention, the qualitative methodology provided a better understanding of the intervention process, and the change initiatives emerging from participatory processes also helped to explain the quantitative results. Additionally, the quantitative methodology also provided evidence for the intervention process and accounted for the changes generated in the employees’ perceptions of their working conditions and psychological well-being. In a similar vein, two studies adapted the RE-AIM (Reach, Effectiveness, Adoption, Implementation, Maintenance) model in a public health context, highlighting four factors that examine both the processes involved in intervention implementation and intervention effectiveness at individual and organizational levels using a wide range of quantitative and qualitative measures (see Estabrook et al., 2012; Jenny et al., 2015).

### Achieving Sustainability and Integration in Standard Procedures

Once the intervention has proven both its effectiveness, or has at least shown that it can be effective following appropriate changes, and its suitability for transfer to other organizational settings, then it is worthwhile making efforts to include it in the organization’s daily routines (i.e., institutionalization), thus rendering it an integral part of standard operating procedures (see also Mayer and Davidson, 2000). In other words, this implementation step refers to the degree to which the intervention or innovation begins to be accepted by and integrated into the organization’s daily procedures.

Despite its theoretical relevance, there are limited studies reporting intervention sustainability in organizational settings. However, some studies have emphasized that intervention maintenance, sustainability and institutionalization are more likely when such programs are aligned with the organizational mission and values and enjoy the support and involvement of several groups and individuals playing a key role within the organization or community (e.g., senior managers, policy makers, community leaders); this has been associated with a greater acceptance toward intervention continuity and obtaining additional resources (Myers et al., 2010; Jenny et al., 2015). In a similar vein, Augustsson et al. (2015) reinforced the development of a health promotion intervention by linking it to an existing continuous improvement system within the organization (Kaizen).

Finally, it is important to promote and disseminate intervention activities through different communication channels (e.g., forums, scientific journals, press releases, technical reports) and share the lessons learned during the design, implementation, and evaluation phases to reach relevant audiences that can become involved and help ensure intervention sustainability.

## Conclusion

Occupational health and safety interventions at an organizational level have been found to improve working conditions and employee health and well-being. However, how to best evaluate the results and effects of such interventions remains a challenging task (e.g., Biron et al., 2010; Kahn-Marshall and Gallant, 2012; Abildgaard et al., 2016). Furthermore, there are several interventions whose design does not include a good follow-up strategy or which lack steering committees aimed at monitoring activity progress and making subsequent decisions regarding the intervention, thus potentially giving rise to misinterpretations of the intervention’s effects. Indeed, implementing an intervention at the organizational level can generate endless problems that, when left unsolved, may lead to a failure to achieve the proposed goals.

Undoubtedly, the main difficulty, and perhaps from which all others derive, lies in maintaining fidelity to the original design during intervention implementation while being able to adapt it to the social reality in which the intervention occurs. Implementation most certainly requires all involved parties to identify and understand the organizational dynamics and processes that render the intervention scenario unique, and will therefore guide the necessary adjustments taken from the original program. Consequently, scholars believe that an evaluation of the implementation process is a prerequisite for being able to explain what actually occurs during the intervention implementation phase and to critically draw conclusions about the intervention outcomes and effects across different levels of analysis (e.g., Dane and Schneider, 1998; Rossi et al., 1999; Nytrø et al., 2000; Nielsen and Randall, 2013).

In response, we proposed some steps including strategic actions that can guide successful intervention implementation. Our aim was not to be exhaustive in our literature review but to use a convenience sample of evidence-based best practices reported in previous literature as examples to help illustrate the proposed implementation steps. Indeed, these studies and evidence-based best practices have described and systematically evaluated the implementation processes behind their interventions. An interesting avenue for further research may be to conduct a systematic literature review while using the implementation steps proposed herein to determine the effectiveness of the implementation process of such interventions reported in the literature over the last decade. In addition, future studies should wider their scope and incorporate findings from other research fields to gain knowledge about the implementation process of interventions at organizational level as a result of cross-fertilization between disciplines (e.g., strategy implementation area: Hitt et al., 2017). Our view is that the implementation process should respect the intervention’s core components given their importance in maintaining intervention quality and effectiveness. In order to identify these components, the intervention should be theory-driven or based on a set of assumptions and mechanisms that indicate how and why the intervention is supposed to achieve intentional changes in organizational settings (e.g., León-Pérez et al., 2016). Simultaneously, implementation also implies a process of adaptation to the particular and changing conditions of the context (i.e., the intervention’s social ecology).

Thus, this paper provides 10 steps that cover a series of key elements at play in intervention implementation, which can be viewed as a cyclical, emergent, and non-linear process that are open to definition depending on the specific problem to be addressed as well as the organization’s characteristics and internal dynamics. In this sense, although our first step in the implementation process was to define the problem, it does not always have to be the first choice action. For example, in risk prevention interventions, the risk assessment phase usually follows on from team building and forming the steering committee. Specifically, in the case of Nielsen et al. (2010a), the authors suggested beginning with a preparation phase where method, structure and culture familiarization are considered necessary. Furthermore, at the early stages, it is important to determine the willingness and readiness of employees and the organization itself to embrace change. As Myers et al. (2010) argued, the starting point of any intervention is the establishment of the mission, values, and rules of interaction between members of the organization.

In short, implementation must be understood as a cyclical and continuous process that encompasses intervention design and evaluation by means of, among other things, problem analysis; selecting and adapting to the context of previous effective intervention strategies; team building and empowerment aimed at strengthening organizational capacity; and monitoring and evaluating the intervention in order to provide information about its impact on the target population. This loop of action and feedback aims to generate knowledge about the changes resulting from the intervention, which, in turn, will initiate a new cycle whereby new problems are identified, and which will serve as the basis for designing new intervention strategies or adaptations of those that have shown to be sustainable over time.

## Author Contributions

All authors equally contributed to the conception of the work and to write and develop the intellectual content of this article. Also, they agreed and approved the final version to be published.

## Conflict of Interest Statement

The authors declare that the research was conducted in the absence of any commercial or financial relationships that could be construed as a potential conflict of interest.
